# Comparative evaluation of post-operative pain following single vs. multiple visit pulpectomy: A clinical assessment

**DOI:** 10.6026/973206300210653

**Published:** 2025-04-30

**Authors:** Jyoti Solanki, Bhagyesh J. Cheta, Arvind Kumar Uikey, Amit Kumar, Anil Raj, Oindrella Ghosh, Pratik Surana

**Affiliations:** 1 Department of Dentistry, Mahamaya Rajkiya Allopathic Medical College Ambedkar Nagar, Uttar Pradesh- 224227, India; 2 Department of Conservative Dentistry and Endodontics, Government Dental College and Hospital Ahmedabad, Gujarat, India; 3 Department of Dentistry, Government Medical College, Seoni, Madhya Pradesh, India; 4 Department of Dentistry, Lord Buddha Koshi Medical College and Hospital, Saharsa, Bihar, India; 5 Department of Public Health Dentistry, Patna Dental College and Hospital, Patna, Bihar, India; 6 Department of Pedodontics and Preventive Dentistry, Kalinga Institute of Dental Sciences, Kalinga Institute of Industrial Technology (KIIT) Deemed to be University, Bhubaneswar, Odisha, India; 7 Department of Pedodontics and Preventive Dentistry, Maitri College of Dentistry and Research Centre, Durg, Chhattisgarh, India

**Keywords:** Pulpectomy, single-visit, multiple-visit, post-operative pain, visual analog scale

## Abstract

Post-operative pain between single-visit and multiple-visit pulpectomy in 50 children (ages 6-10 years) with irreversible pulpitis is compared. Participants were divided into two groups-Group A (single-visit) and Group B (multiple-visit) and pain was assessed using a Visual Analog Scale (VAS) at 6, 24, 48 and 72-hours post-operative. Group A consistently reported significantly lower pain levels (P < 0.05) than Group B. Thus, single-visit pulpectomy reduces post-operative pain more effectively thereby enhancing patient comfort and satisfaction.

## Background:

Maintaining every primary tooth as a fully functional part of the jaw is the prime focus of pulp therapy in deciduous dentition thereby ensuring the space needed for eruption of permanent teeth, efficient mastication, phonation and swallowing and lessens the detrimental psychological possessions associated with missing tooth [1]. To achieve this crucial objective, vital pulp therapy via pulpotomy-defined as the surgical excision of the entire inflamed coronal pulp while maintaining vitality of the radicular pulp within the canals-constitutes the greatest common endorsed method for managing deciduous teeth experiencing reversible pulpitis. Nonetheless, in cases where the radicular canals are irreversibly inflamed or necrotic, a viable pulpotomy is not achievable, thereby requiring either partial or complete pulpectomy [2, 3]. Pulpectomy acts as a traditional treatment method used to prevent early loss of deciduous teeth, which can lead to reduced arch length, insufficient space for the eruption of permanent teeth, impaction of premolars and mesial tipping of adjacent molar teeth following the loss of a deciduous molar [1, 4]. Traditionally, pulpectomy has been performed over multiple visits to ensure thorough cleaning and decontamination of the root canal configuration. However, advancements in endodontic techniques and materials have paved the way for single-visit protocols, raising questions about their efficacy and impact on post-operative discomfort [5, 6- 7]. Figini *et al.* (2008) reported no relevant difference in clinical and radiographic success between single-visit and multiple-visit root canal treatments for permanent teeth [8]. In contrast, Hargreaves *et al.* (2006) and Sathorn *et al.* (2005) argued that single-visit endodontic therapy is superior to multiple-visit approaches in terms of clinical and radiographic outcomes [9, 10]. Hence, an understanding of pain levels associated with different pulpectomy protocols is crucial for making informed decisions that prioritize patient comfort. Therefore, it is of interest to address the gap in knowledge by comparing post-operative pain levels between single and multiple-visit pulpectomy in children aged 6-10 years.

## Materials and Methods:

This research utilized a randomized controlled trial design to assess post-operative pain levels after single-visit versus multiple-visit pulpectomy. The study included 50 children aged between 6 and 10 years, all diagnosed with irreversible pulpitis necessitating pulpectomy. Participants were at random allotted into two groups by implementing a computerized randomization process: Group A (Single-Visit Pulpectomy) with 25 participants and Group B (Multiple-Visit Pulpectomy) also with 25 participants. Inclusion criteria required participants to be within the specified age range, have a clinical and radiographic diagnosis of irreversible pulpitis and be capable of understanding and using the VAS for pain assessment. Exclusion criteria ruled out children with systemic diseases, allergies to medications used in the procedure, or who had previously received pulpectomy in the same tooth.

## Pulpectomy procedure:

Endodontic treatment was done with local anesthesia and rubber dam isolation. The pulp was removed and radiographs determined the working length. Irrigation was performed by 2.5% sodium hypochlorite. In the single-visit group, after drying the canals they were filled with Metapex (*Meta Bio*-Med, Korea) and glass ionomer cement (*Fuji II*, GC, Japan) was used to seal the cavity. In the multiple-visit group, canals were initially filled with calcium hydroxide paste (Prime Dental products, India) and sealed with temporary cement (Orafil-G, Prevest DenPro and India). At subsequent visits, calcium hydroxide was removed, canals dried and obturated with Metapex and cavities sealed with glass ionomer cement.

## Assessment of pain:

Pain intensity following the pulpectomy procedures was measured using the VAS at four post-operative points *i.e.* 6 hours, 24 hours, 48 hours and 72 hours. The VAS, a 10-cm line that ranges from 0 (no pain) to 10 (worst imagined pain), was explained to the participants. They marked their level of pain on the line and scores were recorded and analyzed [11, 12].

## Statistical analysis:

SPSS software version 24.00 was used to do the statistical analysis. T-tests were used to compare the mean VAS scores for both groups at each time interval. Statistical significance was defined as a p-value of less than 0.05.

## Results:

The mean VAS scores for Group A were significantly lower at all-time intervals when compared to Group B (P < 0.05). At 6 hours post-operation, Group A reported an average VAS score of 3.2, whereas Group B reported 4.5. At 24 hours, the scores were 2.1 for Group A and 3.7 for Group B. By 48 hours, Group A averaged 1.5 compared to 2.8 in Group B. At 72 hours, pain scores had decreased to 0.8 in Group A and 1.9 in Group B (Table 1).

## Discussion:

Removing hazardous bacteria and preventing subsequent infections are the primary objectives of performing a pulpectomy on infected deciduous teeth [13]. This helps create an environment conducive to healing the surrounding tissues and alleviates pain and discomfort for young patients. The process of cleaning and shaping the root canal effectively eliminates these microorganisms and can be carried out using two methods: the Single Visit Protocol (SVP) and the Multiple Visit Protocol (MVP) [14]. The MVP method operates on the idea that using an inter-appointment dressing can lower the microbial load in both primary and permanent teeth. While some findings suggest that calcium hydroxide may not consistently lead to sterile root canals and could allow bacteria to regrow, it has been observed that inter-appointment dressing does contribute to a decrease in bacterial counts. However, a reduction in microbial load does not always guarantee healing [14]. SVP for treating primary teeth offers clear benefits, such as its simple procedure and emphasis on efficient root canal cleaning. On the other hand, the MVP demands 3 to 4 appointments, with each one requiring anesthesia, thorough isolation and temporary crown sealing-often leading to complications if the crown comes loose between visits. This not only consumes more time but also increases inconvenience for patients. Additionally, the SVP reduces the number of visits and minimizes radiation exposure, making it even more appealing. Consequently, many experts lean towards the SVP for treating primary teeth [5, 14]. The study provides compelling evidence that single-visit pulpectomy results in significantly lower post-operative pain levels compared to multiple-visit pulpectomy in pediatric patients aged 6-10 years with irreversible pulpitis. This finding has important clinical implications, particularly in improving patient comfort and potentially enhancing the overall treatment experience for young patients, who might otherwise experience anxiety and discomfort associated with multiple dental visits. The consistently lower VAS scores in Group A (single-visit pulpectomy) across all post-operative time points suggest that the single-visit approach not only effectively manages pain but also reduces the duration of post-operative discomfort. This outcome is crucial as post-operative pain can be a significant source of distress and could affect the patient's overall perception of dental care. Several factors could explain the reduced pain associated with single-visit pulpectomy. Completing the procedure in a single visit may reduce the risk of contamination and reinfection, which are potential sources of post-operative pain in multiple-visit treatments. Additionally, eliminating the need for temporary restorations minimizes the risk of microleakage and bacterial ingress, which could further contribute to pain reduction [15]. These findings support the adoption of single-visit pulpectomy as a standard practice for treating irreversible pulpitis in pediatric patients. Not only does this approach improve patient comfort, but it also reduces the overall time and costs associated with multiple visits, benefiting both patients and dental care providers. Signor B and colleagues conducted a qualitative systematic review encompassing 57 studies and three meta-analyses, focusing on postoperative pain following single-visit root canal treatment or vital pulp therapy. They concluded that single-visit procedures are linked to a significantly higher incidence of pain absence and a lower frequency of mild to moderate pain [16]. In a separate systematic review, Kumar and associates assessed postoperative pain occurrences between single-visit and multiple-visit root canal therapies, finding that most studies reported no significant differences in pain levels as measured by the Visual Analogue Scale. However, few studies indicated that pain post-obturation was less common in single-appointment treatments compared to multi-visit ones [17]. Additionally, Prashanth MB and colleagues conducted an in vivo study to examine postoperative outcomes for single-visit versus multiple-visit endodontic therapy in both vital and non-vital teeth. Their results showed no significant differences in success rates, postoperative pain, tenderness, or swelling between the two approaches. Collectively, these studies suggest that single-visit endodontic therapy may be effectively incorporated into regular dental practice [18]. Despite the positive outcomes, the study has some limitations. Despite being sufficient for preliminary results, 50 participants sample size might not be sufficient to extrapolate findings to larger groups. Additionally, the study's age range might limit its applicability to different pediatric age groups or adult patients. In order to reinforce these results, large and more varied sample sizes should be the goal of future research. Sevekar & Gowda (2017) also suggest that more extensive research should be conducted to evaluate the impact of various instrument systems, obturating materials, irrigating solutions and patient cooperation on the incidence of postoperative pain in primary teeth. Additionally, there is a need to identify the risk factors associated with post-endodontic pain and flare-ups in primary teeth [15].

## Conclusion:

Single-visit pulpectomy emerges is a superior approach of managing post-operative pain. This suggests a shift in pediatric dental practice towards single-visit procedures wherever clinically feasible.

## Figures and Tables

**Table 1:** Comparison of mean VAS score at different time interval

**Interval**	**Groups**	**Mean VAS Score**	**t value**	**p value**
After 6 hours	I	3.2 ± 0.15	1.01	<0.05*
	II	4.5 ± 0.20		
After 24 hours	I	2.1 ± 0.22	1.15	
	II	3.7 ± 0.18		<0.05*
After 48 hours	I	1.5 ± 0.16	1.56	
	II	2.8 ± 0.15		<0.05*
After 72 hours	I	0.8 ± 0.10	1.32	<0.05*
	II	1.9 ± 0.10		
*Significant
